# Instance of Recovery from a Large Dose of Laudanum, by Active Remedies

**Published:** 1814-06

**Authors:** John C. Yeatman

**Affiliations:** Member of the Royal College of Surgeons. Bristol, College Green


*For the Medical and Physical Journal.*

### *Instance of Recovery from a large Dose of Laudanum, by active Remedies; by* Mr. J. C. Yeatman.

THE following important case has lately come under my care.

On the 15th of April, Mr. J. B., æt. 21, of the sanguineous temperament, swallowed, between the hours of two and three P. M., an ounce of laudanum, with the view to destroy himself. He repaired immediately to the house of a friend and neighbour, where he ate a plentiful dinner: while at his meal the lady of the house observed a dullness of the eyes, but not suspecting the cause, took no further notice of it. After dinner he called upon another friend, when he became alarmingly ill, and said he had taken laudanum. At half-past five I was sent for, and on being informed of the nature of the case, I put a lump of Sulphas Zinci in my pocket, and hastened to the poisoned youth. I found my patient laying back in an arm-chair totally insensible; a death-like paleness had come on, and his features were distorted, particularly the angles of the mouth; the lower jaw was fixed; respiration was performed at long intervals, and laboriously; the pupils were not obedient to the stimulus of light; the skin was cold; the hands clenched; the pulse rapid, small, and intermittent. I dissolved thirty grains of the Sulphate of Zinc in half a pint of water, forced open his mouth, and poured it all into the stomach. We shook him well in the chair, and having dragged him through the passage, from the parlour to the kitchen, succeeded in rousing him. His father had now arrived, and being a man of a firm mind, although greatly afflicted at the occurrence, proved under my direction an able assistant. I desired him to force his hand into the mouth, and to thrust his finger to the fauces, which provoked immediate vomiting. The smell of laudanum was perceptible. The vomiting, however, was not so copious as the emergency of the case required, and I gave him twenty grains more of the Sulphate of Zinc, which, by by frequently irritating the fauces, and by the assistance of warm water, made him discharge a quart of the contents of the stomach in less than fifteen minutes after my arrival. I also gave four ounces of olive oil, which increased the nausea, and rendered the vomiting less laborious. We shook him repeatedly, and between two persons had him lugged up and down the room. He was now enabled to move one leg before the other, a perspiration broke out upon his fore-head, and he had power to groan aloud. A large draught of acetic acid, slightly diluted, increased the sensibility of the stomach. He became affected with spasm, particularly of the muscles of the back, neck, and extremities. While other means than those already resorted to were in preparation, I had him jolted in a carriage to his father's residence, and on being taken out he again evinced an inclination to vomit, which I encouraged by giving a table-spoonful of mustard mixed in a tumbler glassful of diluted acetic acid. This remedy, while it roused him, considerably excited active nausea, and about a second quart of the contents of the stomach was ejected.

I now felt fully satisfied on that point: still, however, my patient required the most active means to rouse him from occasional stupor and lethargy. Four blisters were immediately applied to different parts of the body, and mustard cataplasms to the palms. The citric and acetic acids were administered. The citric acid I gave in combination with a solution of subcarbonate of potass during effervescence. We did not cease walking him every five minutes up and down the room. The pulse became stronger and more regular, and the pupils readily contracted to the light of a candle. He became violently affected, at shorter intervals, with spasm, and would often throw his head, neck, and upper part of the trunk suddenly and forcibly backwards, keeping himself in that position for a few moments. At length he uttered a monosyllable—soon afterwards called out the name of a friend—became hysterical—recognised his mother-in-law, and eagerly embraced her. He now grew more and more rational and tranquil, when I left him to the care of his relations at eleven o'clock, giving them strict injunctions to keep him upon the move during the night, and directing them to administer ten grains of the citric acid in two table-spoonfuls of the solution of subcarbonate of potass every two hours. In the morning I found my patient in a bed, where he had been permitted to sleep for two or three hours. His face was flushed and hot; lips dry; tongue lined with a brown-coloured fur; throat and stomach sore; pain in the head, with a sense of weight in the eyes; was very drowsy; drowsy; pulse quick, but not hard nor full. I ordered him to be dressed and roused, some strong coffee to be given him, acidulous drinks to be taken in the course of the day, and the whole of the following mixture:

R. Ol. Ricini, ℥ij.

Vitell. Ovi, q. s.

Aq. Menth. Pip. ℥iiiſs.

Syr. Aurant. ℥ſs. M. f. Mistura.

In the evening his pulse was 89, and a little fuller. He merely complained of slight heat and thirst, with soreness of the throat and stomach. The mixture had occasioned two scanty evacuations.

R. Magnes. Sulphat. ℥j. solve in

Infus. Rosæ, ℥xij. & adde

Syr. Rhæados, ℥iſs.

Cras primo mane sumend.

I also ordered him to sip a little of the Almond Emulsion after, and to drink plentifully of Decoctum Hordei. The next morning he had several stools. He was permitted to sleep some few hours on the second and third nights, and in a few days he entirely recovered.

It will be observed, that although three hours elapsed between the taking of the poison and the exhibition of the remedies, success crowned our exertions; and this and other cases of a similar nature, give us reason to conclude that the most desperate of their kind will happily yield to prompt, vigorous, and persevering treatment.

The hearty dinner and the exercise immediately after it no doubt in some measure retarded the operation, and perhaps lessened the effects, of the laudanum; but three hours was certainly an ominous, and had nearly proved a fatal, interim.

In the first instance I was under great apprehension lest the narcotic property of the opium had destroyed the sensibility of the stomach, and therefore I could give but little hope of a recovery—a consideration, however, which only tended to increase my exertions.

We are told that two indications are to be fulfilled in cases of the foregoing kind.

1st, To occasion a speedy evacuation of the stomach; and 2dly, to counteract the effects of the opium.

It may, perhaps, be questioned whether the Sulphate of Zinc did any good in the case before us, as the effects of the laudanum had taken place before its exhibition. Dr. Cullen, when speaking of the treatment of that kind of apoplexy occasioned by opium, alcohol, and other narcotic poisons, says, "If the poison has been taken into the stomach long before before its effects have appeared, we judge that upon their appearance the exciting of vomiting will be useless, and may perhaps be hurtful." I should not, however, think it safe to suffer the poison to remain in the stomach, as the danger of the patient may increase with the presence of the narcotic, and as we cannot with certainty determine when it has exerted its fullest effects on the animal system. The emetic also acts as a powerful stimulus, since the patient is greatly roused by the exertion of vomiting.

The remedies employed in the second indication appear to prove salutary by increasing the sensibility of the stomach, by rousing the energy of the nerves, and through their medium by restoring the functions of the brain and of the lungs. We may not be acquainted with the modus operandi of the vegetable acids in these cases, and this still remains an interesting and important subject for accurate investigation.

I would remark that I have in my recollection two cases in which the use of the lancet proved fatal. In the one, which was treated by a physician of Philadelphia, the temporal artery was opened soon after a slight re action had taken place; in the other, which was at a public hospital, the jugular vein was punctured whilst the patient was labouring under the narcotic effects of laudanum. As, however, the observations I have thought it necessary to make would at present occupy too much of your valuable Journal, I will take another opportunity of requesting their insertion.*

JOHN C. YEATMAN,

*Member of the Royal College of Surgeons.*

*Bristol*, *College Green*,
*May* 10, 1814.

* The Editors will feel themselves obliged by Mr. Yeatman's early attention to this interesting subject.

